# Exploring the Bioactive Properties and Therapeutic Benefits of Pear Pomace

**DOI:** 10.3390/antiox13070784

**Published:** 2024-06-28

**Authors:** Joana Ferreira, Karolina Tkacz, Igor Piotr Turkiewicz, Isabel Santos, Mariana Camoesas e Silva, Ana Lima, Isabel Sousa

**Affiliations:** 1 LEAF—Linking Landscape, Environment, Agriculture and Food—Research Center, Associate Laboratory TERRA, Instituto Superior de Agronomia, Universidade de Lisboa, Tapada da Ajuda, 1349-017 Lisboa, Portugal; isabelsousa@isa.ulisboa.pt; 2 Department of Fruit, Vegetable and Plant Nutraceutical Technology, Faculty of Biotechnology and Food Science, Wrocław University of Environmental and Life Sciences, 37 Chełmońskiego Street, 51-630 Wrocław, Poland; karolina.tkacz@upwr.edu.pl (K.T.); igor.turkiewicz@upwr.edu.pl (I.P.T.); 3 Veterinary and Animal Research Centre (CECAV), Faculty of Veterinary Medicine, Lusófona University, 376 Campo Grande, 1749-024 Lisboa, Portugal; p2619@ulusofona.pt (I.S.); agusmaolima@gmail.com (A.L.); 4 Faculty of Veterinary Medicine, Lusófona University, 376 Campo Grande, 1749-024 Lisboa, Portugal; mrcamoesas19@gmail.com

**Keywords:** cholinesterase, MMP-9, phenolics, tocols, carotenoids, xanthophylls, procyanidins, amino acids, AAT, chromatography

## Abstract

The fruit juice industry generates a significant amount of waste, with a strong impact on the environment and the economy. Therefore, researchers have been focusing on the characterization of resources considered as food waste. This work provides information about the lipophilic and polar metabolites of pear pomace flours (PPFs) as a tool that can shed more light on the bioactive potential of this residue. Using UPLC-PDA, UPLC-FLR, and GC-MS, the study identified and quantified PPF’s polar and non-polar metabolites. Essential, conditional, and non-essential amino acids were found, with asparagine being the most abundant. Isoprenoids, including lutein, zeaxanthin, and carotene isomers, ranged from 10.8 to 22.9 mg/100 g dw. Total flavonoids and phenolic compounds were 520.5–636.4 mg/100 g dw and 536.9–660.1 mg/100 g dw, respectively. Tocotrienols and tocopherols were identified, with concentrations of 173.1–347.0 mg/100 g dw and 468.7–913.4 mg/100 g dw. Fatty acids were the major non-polar compounds. All fractions significantly reduced matrix metalloproteinase-9 (MMP-9) activity. Although PPF had lower antioxidant potential (3–6 mmol Trolox/100 g dw), it inhibited AChE and BuChE by 23–30% compared to physostigmine salicylate. These findings suggest that pear pomace waste can be repurposed into functional products with valuable bioactive properties by re-introducing it in the food chain.

## 1. Introduction

Sustainability and waste minimization are crucial principles guiding the modern food industry. As the quantity of food waste continues to grow, researchers are driven to discover innovative solutions to recover valuable components. By-products from the fruit industry, such as apple and pear pomaces resulting from juice pressing, hold immense potential as a source of bioactive compounds, including polyphenols [1,2]. These compounds play a significant role as antioxidants and free radical scavengers and exhibit various health benefits, such as antidiabetic, antitumor, antihypertensive, anti-inflammatory, antiaging, cardio-protective, and neuroprotective properties [3]. Thus, it becomes imperative to valorize and utilize these waste materials effectively.

Pear pomace, a by-product derived from pear processing, contains a range of bioactive compounds, including carbohydrates, amino acids, vitamins, minerals, and fibers that hold potential for industrial applications. Researchers have proposed several uses for pear pomace, such as the coproduction of bacterial cellulose and pear vinegar [4] and as a source of fiber and antioxidants to combat obesity and oxidative stress in animal studies [5,6,7]. Notably, the phenolic bioactive molecules are most abundant in the seeds and peels of pears, making pear pomace an excellent source of these compounds, as pear pulps contain lower concentrations [8].

Numerous studies have characterized the bioactive molecules present in pears, revealing the presence of polyphenols like phenolic acids, flavonoids, and triterpenes [9]. In-depth chemical analysis conducted by Li and co-workers [10] demonstrated that the prevailing monomeric compounds in both the peel and pulp of ten pear varieties were arbutin, oleanolic acid, ursolic acid, chlorogenic acid, epicatechin, and rutin. Additionally, the quantification of total phenols, flavonoids, and triterpenes revealed significantly higher concentrations in the peel compared to the pulp [10], underscoring that a considerable amount of bioactive compounds are discarded in pear pomace along the pear-processing food chain.

The interest in studying these by-products is continually rising, given that the apple and pear juice industry generates numerous by-products rich in various bioactive components, mainly phenolic compounds. These bioactive substances have diverse applications in medicine, cosmetics, and the food industry. Particularly in the food sector, where sustainable and functional foods are gaining popularity, the abundance of fruit juice industry by-products presenting multiple bioactive compounds has become a valuable resource.

MMP-9 is an enzyme involved in the degradation of extracellular matrix components, playing a critical role in tissue remodeling, inflammation, and cellular migration [11,12]. Dysregulated MMP-9 activity has been implicated in various pathological conditions, including cancer, chronic inflammatory diseases, and aging-related disorders [11,12,13]. In inflammatory diseases, excessive MMP-9 activity can exacerbate tissue damage and inflammatory responses. Moreover, in the context of aging, increased MMP-9 activity is associated with the deterioration of tissue structure and function [14]. Consequently, MMP-9 is considered a valuable therapeutic target [15]. Under this context, nutraceuticals and functional foods containing MMP-9 inhibitory compounds can provide a non-invasive preventative strategy to mitigate the enzyme’s detrimental effects and have become a hot topic in research. 

Phenolic compounds, which are abundant in fruits, have shown significant MMP-9 inhibitory activity. For instance, flavonoids and other polyphenols present in various fruits have been reported to inhibit MMP-9 activity effectively [16,17]. Pears, in particular, contain a variety of phenolic compounds, including flavonoids, phenolic acids, and tannins, which possess potential MMP-9 inhibitory effects. Pear pomace, a byproduct of pear juice production, is particularly rich in these bioactive compounds due to its concentration of peel and pulp remnants, which are known to have higher phenolic content than fruit juice itself. This concentration of bioactive compounds might enhance its potential as a functional food ingredient with significant health benefits and provide novel insights into its usefulness as a functional food against cancer, inflammatory diseases, and age-related tissue degeneration [18,19].

## 2. Materials and Methods

### 2.1. Reagents and Standards

Potassium hydroxide, hydrochloric acid, and acetic acid were purchased from Honeywell (Charlotte, NC, USA). Sodium hydroxide, tetrahydrofuran (THF), ascorbic acid, and all-*trans*-β-carotene were acquired from Fisher Scientific Chemicals (Waltham, MA, USA). Bis(trimethylsilyl)trifluoroacetamide (BSTFA) was sourced from TCI (Chuo-Ku, Tokyo, Japan). Methanol (analytical grade), formic acid, acetonitrile, acetone, ethanol, hexane, ethyl acetate, butylhydroxytoluene (BHT), magnesium carbonate, magnesium hydroxide, α-carotene, all-*trans*-lutein, all-*trans*-β-cryptoxanthin, (−)-epicatechin, (+)-catechin, procyanidin B2 and C1, chlorogenic acid, 3,5-di-caffeoylquinic acid, quercetin, kaempferol-3-*O*-glucoside, kaempferol-3-*O*-rutinoside, dichloromethane, ammonium chloride, and pyridine were obtained from Sigma–Aldrich (St. Louis, MO, USA). All other reagents were also sourced from Sigma–Aldrich (St. Louis, MO, USA).

### 2.2. Sample Preparation

The pear pomace flour (PPF) from *Pyrus communis* L., var. Rocha, was sourced from ALITEC—Alimentos Tecnológicos SA (Nazaré, Portugal). Prior to the acquisition, it underwent a convection drying tunnel process (Tecnofruta, Valencia, Spain) under specified conditions (80–85 °C, 110 min, 55 Hz air flow speed) followed by a grinding process (Ferneto, Vagos, Portugal).

The average laboratory sample was fractionated through sieves with the following mesh sizes: 710 µm, 180 µm, 75 µm, 53 µm. Each fraction was weighed to determine its percentage (710 µm—14.7%, 180 µm—55.0%, 75 µm—20.3%, 53 µm—6.7%) and then subjected to an extraction process. 

### 2.3. Antioxidant Capacity

The extraction procedure for assessing biological activities was previously described by Wojdyło and co-workers [20]. In brief, the samples were homogenized with 6 mL of 80% methanol (*v*/*v*) and sonicated twice for 20 min, with a 24-h interval between sonications (Sonic 6D, Polsonic, Warsaw, Poland). The solution was then centrifuged at 15,000× *g* for 10 min using an MPW-352R (MPW Med. Instruments, Warsaw, Poland), and the supernatant was filtered through a Minispike Syringe Filter (PTFE, 0.20 μm, Waters Corp., Milford, CT, USA) for analysis. Antioxidant capacity was evaluated using spectrophotometric (2,2-azino-*bis*-3-ethylbenzothiazoline-6-sulphonic acid, or ABTS) and spectrofluorometric (oxygen radical absorbance capacity, or ORAC) methods, as described by Nowicka and co-workers [21]. Using 2,2′-azino-bis(3-ethylbenzothiazoline-6-sulfonic acid) (ABTS), a radical cation can be formed that absorbs light at 734 nm in water. When antioxidants are introduced into a solution containing the ABTS radical cation, the absorption decreases because the radical cation is neutralized [22]. Another method for screening antioxidants is the oxygen radical absorbance capacity (ORAC) assay. This assay involves an oxygen radical-generating compound, 2,2′-azobis(2-amidinopropane)dihydrochloride, which produces reactive oxygen species (ROS) that oxidize fluorescein, a highly fluorescent compound. The oxidation product of fluorescein has very weak fluorescence; therefore, the fluorescence of the solution diminishes over time as the ROS oxidizes the fluorescein. When an antioxidant is added, it reacts with the ROS, delaying the oxidation of fluorescein until the antioxidant is exhausted [23]. Results were reported as the mean of three measurements ± SD and expressed in mmol Trolox per 100 g of dry weight.

### 2.4. AChE and BuChE Inhibitory Activity

Anti-aging activity was evaluated by measuring acetylcholinesterase (AchE) and butylcholinesterase (BuChE) inhibition, as previously described by Tkacz and co-workers [24]. Results, expressed as percentage inhibition at concentrations of 100 mg/mL for both anti-AchE and anti-BuChE effects, represent the mean of three measurements ± SD. All measurements were performed using a Synergy™ H1 multi-mode microplate reader (BioTek, Winooski, VT, USA). Physostigmine salicylate was used as a positive control drug.

### 2.5. MMP-9 Inhibitory Activity

MMP-9 activity was measured using the Human MMP-9 ELISA assay kit (Eagle Biosciences, Nashua, NH, USA). In brief, 100 μL of each sample was added to a commercial MMP-9 standard at a concentration of 250 pg/mL and incubated for 1 h to allow for enzymatic inhibition. All samples were then processed according to the manufacturer’s instructions, and absorbance was read at 450 nm using a microplate reader (LB942 TRISTAR 3).

### 2.6. Identification and Quantification of Free Amino Acids

The extraction procedure for the UPLC-PDA/MS analysis of free amino acids was previously described by Collado-González and co-workers [25]. A total of 80 mg of dry sample was extracted twice using shaking for 30 min on a rotator (Rotator Multi RS-60, Biosan, Riga, Latvia) and 30 min of ultrasound treatment (Sonorex RK 100 H, Bandelin, Berlin, Germany) with 1.5 mL of a methanol:water mixture (50:50, *v*/*v*). The samples were then centrifuged (14,000 rpm for 7 min at 20 °C), and the supernatant was used for derivatization. Identification and quantification were performed using an ACQUITY Ultra Performance Liquid Chromatography (UPLC) system equipped with a photodiode array (PDA) detector (Waters Corp., Milford, MA, USA) and a G2 Q/TOF mass spectrometer (MS) (Waters, Manchester, UK) with an electrospray ionization (ESI) source operating in positive mode. The exact separation parameters and MS settings were previously described by Wojdyło et al. [26]. Separations of individual amino acids were carried out using an AccQ Tag Ultra BEH column (2.1 × 100 mm, 1.7 μm) (Waters Corp.; Milford, MA, USA). The column was kept at 50 °C, the samples at 10 °C. The injection volume was 3 μL and the elution was performed at a flow rate of 0.50 mL/min. The mobile phase consisted of solvent A (50 mL of solution: acetonitrile, formic acid, and 5 mM ammonium acetate in water (10:6:84, *v*/*v*/*v*) in 950 mL water) and solvent B (acetonitrile and formic acid; 99.9:0.1, *v*/*v*). The gradient profile was 99.0% A at 0–0.30 min, 97.0% A at 3.20 min, 88.0% A at 6.80 min, 82.0% A at 8.95 min, 74.0% A at 9.50 min, 67% A at 9.80 min, 40.0% A at 10.65 min, and 99.0% A at 14.50–15.00 min. The PDA spectra for amino acids were measured at a wavelength of 260 nm. Retention times and spectra were compared to authentic amino acid standards. Results were expressed as mg per 100 g of dry weight.

### 2.7. Identification and Quantification of Carotenoids

The extraction procedure and conditions for the UPLC-PDA analysis of carotenoids were previously described by Tkacz et al. [27]. Briefly, the dried samples (about 0.5 g) were vortexed in the dark at 300 rpm for 30 min (DOS-10L Digital Orbital Shaker, Elmi Ltd.; Riga, Latvia) with 5 mL of a methanol:acetone:hexane (1:1:2, *v*/*v*/*v*) mixture. To prevent oxidation, 10% (*m*/*v*) 4MgCO_3_ × Mg(OH)_2_ × 5H_2_O was added. The extraction process of the solid residue was repeated four times, with each cycle including centrifugation (4 °C, 10 min at 15,000 rpm; MPW-352R; Warsaw, Poland) and the collection of the supernatants, which were finally evaporated to dryness (XCV–5400 XcelVap^®^ Evaporation System, Horizon Technology, Inc.; Salem, MA, USA). The pellet was diluted with 1 mL of methanol:THF (4:1, *v*/*v*). The solutions were filtered through a Minispike Syringe Filter (PTFE, 0.20 μm, Waters Corp.; Milford, MA, USA) and used for analysis. The analysis of carotenoids was performed using an ACQUITY UPLC system with a PDA detector (Waters Corp., Milford, MA, USA). Separations of carotenoids and xanthophylls were carried out using an ACQUITY UPLC BEH Shield RP18 column (2.1 × 100 mm, 1.7 μm) (Waters Corp.; Milford, MA, USA). The column was kept at 32 °C, and the samples at 10 °C. The injection volume was 10 μL and the elution was performed at a flow rate of 0.50 mL/min. The mobile phase consisted of solvent A (water and formic acid; 99.9:0.1, *v*/*v*) and solvent B (acetonitrile and methanol; 70:30, *v*/*v*). The gradient profile was 25.0% A at 0–0.60 min, 4.90% A at 6.50 min, 0.0% A at 13.60 min, and 25.0% A at 14.60–16.60 min. The PDA spectra were measured at wavelength 450 nm. Qualitative determination of carotenoids was carried out using UV-VIS spectra and retention times of reference compounds. Calibration curves were created for all-*trans*-β-carotene, α-carotene, all-*trans*-lutein, and all-*trans*-β-cryptoxanthin. Results were expressed as mg per 100 g of dry weight. 

### 2.8. Analysis of Tocopherols (TF) and Tocotrienols (TT)

The extraction procedure and conditions for the UPLC-FLR analysis of tocopherols and tocotrienols were previously described by Turkiewicz [28]. Briefly, the samples (about 1 g) were homogenized with 5 mL of ethanol containing 0.05% BHT (*m*/*v*). Saponification was carried out with 1 mL of 60% KOH at 80 °C for 60 min. The samples were then mixed with 10 mL of hexane:ethyl acetate (9:1, *v*/*v*) containing 0.05% BHT (*m*/*v*). After 30 min, a saturated NaCl solution was added. The upper layer was collected, evaporated to dryness, and dissolved in 1 mL of methanol:THF (4:1, *v*/*v*). The solutions were filtered through a Minispike Syringe Filter (PTFE, 0.20 μm, Waters Corp., Milford, MA, USA) and used for analysis. Tocopherols and tocotrienols were analyzed using an ACQUITY UPLC system with a fluorescence (FLR) detector (Waters Corp., Milford, MA, USA). Separations of individual tocopherols and tocotrienols were carried out using an ACQUITY UPLC BEH Shield RP18 column (2.1 × 100 mm, 1.7 μm) (Waters Corp.; Milford, MA, USA). The column was kept at 30 °C, the samples at 10 °C. The injection volume was 5 μL and the elution was performed at a flow rate of 0.45 mL/min. The mobile phase consisted of solvent A (methanol) and solvent B (water). The gradient profile was 88.0% A at 0–12.0 min. The wavelengths of excitation/emission were 290/330 nm. Compounds were identified and quantified using reference standards of α-, β-, γ-, and δ-tocopherols and tocotrienols. Results were expressed as μg per 100 g of dry weight.

### 2.9. Identification and Quantification of Phenolic Compounds and Polymeric Procyanidins Using UPLC-PDA-FLR Methods

The extraction procedure for the UPLC-PDA analysis of phenolic compounds was as follows: First, powders (about 0.4 g) were weighed and thoroughly mixed with 6 mL of methanol:water (30:70, *v*/*v*), followed by the addition of acetic acid (1%, *v*/*v*) and ascorbic acid (1%, *m*/*v*). The mixtures were sonicated for 15 min, stored at 4 °C for 20 h, and then sonicated again for 15 min. Subsequently, the supernatant was collected after centrifugation, and the resulting extracts were filtered through a Minispike Syringe Filter (PTFE, 0.20 μm, Waters Corp.; Milford, MA, USA) for analysis.

Quantitative analysis of polyphenols (flavan-3-ols, flavonols, phenolic acids) was performed using UPLC-PDA, following the method described by Turkiewicz et al. [29].

Separations of individual phenolics were carried out using an ACQUITY UPLC BEH C18 column (2.1 × 100 mm, 1.7 μm) (Waters Corp.; Milford, MA, USA). The column was kept at 30 °C and the samples at 10 °C. The injection volume was 5 μL and the elution was performed at a flow rate of 0.42 mL/min. The mobile phase consisted of solvent A (water and formic acid; 99.9:0.1, *v*/*v*) and solvent B (acetonitrile and formic acid; 99.9:0.1, *v*/*v*). The gradient profile was 99.0% A at 0 min, 65.0% A at 12.0 min, 99.0% A at 12.30 min, and 99.0% A at 12.3–15.0 min. Detection was conducted at specific wavelengths: flavan-3-ols at 280 nm, phenolic acids at 320 nm, and flavonols at 360 nm. Retention times and spectra were compared with those of pure standards, and calibration curves were generated from (−)-epicatechin, (+)-catechin, procyanidin B2 and C1, chlorogenic acid, 3,5-di-caffeoylquinic acid, quercetin, kaempferol-3-*O*-glucoside, and -3-*O*-rutinoside.

Analysis of polymeric procyanidins was conducted using the phloroglucinolysis method described by Wojdyło et al. [20]. Next, 50 mg of the dried sample was weighed in a 2 mL tube; then, 0.8 mL of the methanolic solution of phloroglucinol (75 g/L) and ascorbic acid (15 g/L) was added. After the addition of 0.4 mL of methanolic HCl (0.3 mol/L), the vials were closed and incubated for 30 min at 50 °C with continuous vortexing using a thermo shaker (ThermoMixer^®^ C, Eppendorf, Hamburg, Germany). The reaction was stopped by placing the vials in an ice bath by drawing 0.6 mL of the reaction medium and diluting with 0.6 mL of 0.2 mol/L sodium acetate buffer. Next, the vials were cooled in ice water and centrifuged immediately at 14,000 rpm for 7 min at 20 °C.

Separations of polymeric procyanidins were carried out using an ACQUITY UPLC BEH Shield RP18 column (2.1 × 50 mm, 1.7 μm) (Waters Corp.; Milford, MA, USA). The column was kept at 15 °C and the samples at 10 °C. The injection volume was 5 μL and the elution was performed at a flow rate of 0.50 mL/min. The mobile phase consisted of solvent A (water and acetic acid; 97.5:2.5, *v*/*v*) and solvent B (acetonitrile). The gradient profile was 98.0% A at 0–0.60 min, 97.0% A at 2.20 min, 90.0% A at 5.0 min, 85.0% A at 6.0 min, and 98.0% A at 7.5 min. The wavelengths of excitation/emission were 278/360 nm. Results were expressed as mg per 100 g of dry weight.

### 2.10. Lipophilic Extracts Composition Using GC-MS

The extraction procedure for assessing the lipophilic composition was previously described by Ferreira and co-workers [30]. Briefly, the extraction with dichloromethane was performed in a Soxhlet apparatus for 6 h. Portions of the dichloromethane extracts (5 mL) were evaporated under nitrogen flow and then vacuum-dried at room temperature. For analysis, the samples were derivatized by dissolving the residues in 150 μL of pyridine and adding 100 μL of bis(trimethylsilyl)trifluoroacetamide (BSTFA) to convert the hydroxyl and carboxyl groups into trimethylsilyl (TMS) ethers and esters. The mixture was heated at 60 °C for 3 h. The derivatized extracts (1 μL) were then analyzed using GC-MS (Thermo Scientific Trace 1300 Gas-Chromatograph coupled with a Mass Spectrometer, Waltham, MA, USA), operating with an ionization energy of 70 eV and maintaining the MS source at 230 °C. The GC conditions were as follows: DB-1 capillary column (30 m × 0.25 mm × 0.25 μm film thickness), injector at 220 °C. The column temperature was initially held at 50 °C for 1 min then ramped to 150 °C at a rate of 10 °C per minute, followed by ramps to 300 °C at 4 °C per minute, to 370 °C at 5 °C per minute, and, finally, to 380 °C at 8 °C per minute, with an isothermal period of 5 min.

Identification of compounds as TMS derivatives was accomplished by comparing their mass spectra with a GC-MS spectral library (Wiley: Hoboken, NJ, USA; NIST) and their fragmentation profiles with published data. Peak areas in the total ion chromatogram (TIC) were determined and expressed as normalized relative percentages. The calculated composition was semi-quantitative/qualitative due to the absence of co-injected standards for each chemical family and undetermined response factors. Each aliquot was injected in triplicate. 

### 2.11. Statistical Analysis

Statistical analysis of the experimental data was performed using SPSS (version 29, IBM, Armonk, NY, USA) through variance analysis (one-way ANOVA) and by the Tukey test as the post hoc at a significance level of 95% (*p* < 0.05). All results have been presented as average (*n* = 3) ± standard deviation.

## 3. Results

### 3.1. Antioxidant Capacity

The antioxidant capacity of pear pomace extracts was assessed as oxygen radical absorbance capacity (ORAC) and free radical-scavenging activity (ABTS) (Table 1). The antioxidant activity showed higher values in the ORAC test, between 5.3 and 6.1 mmol Trolox/100 g dw (dry weight), while the antioxidant activities in ABTS ranged from 2.3 to 3.0 mmol Trolox/100 g dw. This difference is expected since the two methods are based on different principles and provide slightly different insights into the antioxidant activity of the tested samples. While ORAC measures the ability of a substance to neutralize peroxyl and hydroxyl radicals generated during the assay to mimic the oxidative stress conditions in the body caused by free radicals, the ABTS measures the ability of a substance to scavenge the ABTS^•+^ radical cation formed by the reaction of ABTS with potassium persulfate [31]. For comparison, in the studies by Wan and co-workers, for a selected fresh pear fruit cultivar, the values of ORAC antioxidant activity were much lower (0.75 mmol/100 g dw), almost eight-fold lower than the range of 5.3–6.1 mmol Trolox/100 g dw [32]. 

The results from ABTS show that the antioxidant potential is higher for particles with higher particle size (over 710 µm), mostly constituted by peels, seeds, and core, ranging from 2.3 to 3.0 mmol Trolox/100 g dw, although not statistically different. The antioxidant activity results from ORAC follow the same trend, although no statistical differences are observed.

### 3.2. AChE and BuChE Inhibitory Activity

**Figure 1:**
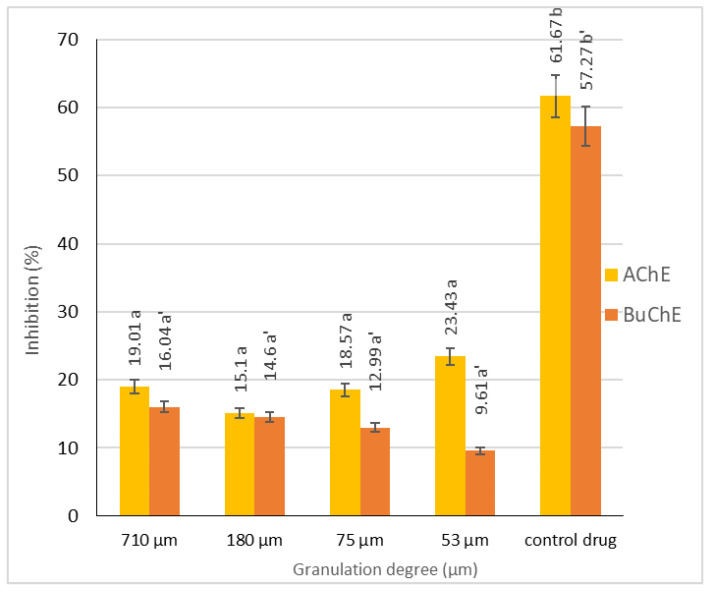
AChE and BuChE inhibition activity (%) of the different PPF fractions. The data shown are mean values (*n* = 3) followed by an alphabet letter (for comparison between the different granulometric fractions). Different letters mean significantly different results (Tukey’s HSD; *p* ≤ 0.05).

Cholinesterase inhibitors play a significant role in the treatment of neurodegenerative diseases, as they help inhibit the progression of lesions with relatively low side effects. However, it is important to note that while these inhibitors can provide therapeutic benefits, they do not completely eliminate the underlying causes of the diseases [33]. The activity of PPF against acetyl- and butylcholinesterase (AChE and BuChE, respectively) as a potential way to inhibit degenerative changes by increasing transmission in the cholinergic system was investigated (Figure 1). 

The anticholinergic activity of different granulometric fractions of PPF was examined as the ability to inhibit AChE and BuChE. The results are shown as percentage inhibition at a concentration of 100 mg/mL enzyme (Figure 1). These enzymes are involved in the breakdown of the neurotransmitter acetylcholine, a low level of which is typical for incurable and progressive Alzheimer’s disease, dementia, and many other neurodegenerative disorders. The anti-AChE and anti-BuChE activities, as percentage inhibition, fluctuated from 15.1 to 23.4% for AchE and from 9.6 to 24.7% for BuChE, which is considerably low when compared to the literature for different pear cultivars (40–80% for AchE and 9–75% for BuChE) [34]. 

### 3.3. MMP-9 Inhibitory Activity

**Figure 2:**
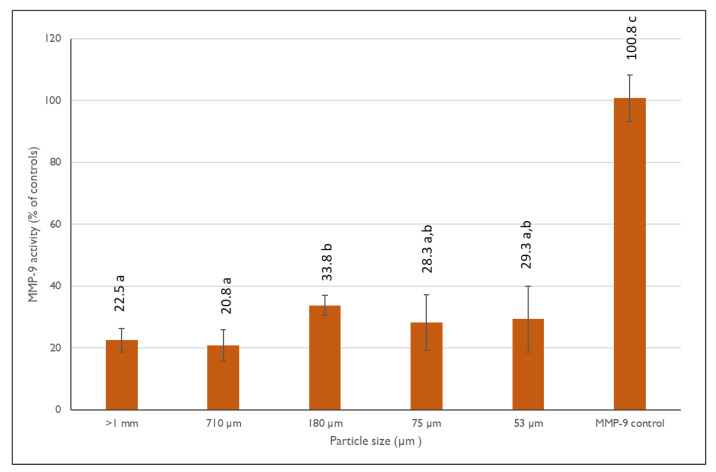
MMP-9 inhibitory activity as a percentage (%) of controls for the different PPF fractions. The data shown are mean values (*n* = 3) followed by an alphabet letter (for comparison between the different granulometric fractions). Different letters mean significantly different results (Tukey’s HSD; *p* ≤ 0.05).

In this study, we evaluated the inhibitory effects of various fractions of pear pomace with different granulometries on MMP-9 activity (Figure 2). Each fraction was tested in triplicate, and results were compared to both positive and negative controls. 

Results show that all pear pomace fractions were able to significantly impair MMP-9 activity (*p*  <  0.001), reducing gelatinolytic activity by more than 50% in all particle sizes. Notably, while all fractions showed substantial inhibition, there were no statistically significant differences among the different granulometries (*p* > 0.05), suggesting that the inhibitory compounds present in the pear pomace are effective regardless of the particle size of the pomace fractions.

Our findings align with previous studies that have highlighted the potential of fruit-derived phenolic compounds in inhibiting MMP-9 activity. For instance, blueberries, grapes, and persimmon have been reported to contain phenolic acids and flavonoids that exhibit strong MMP-9 inhibitory effects [16,17,35].

Pear pomace, a byproduct of pear juice production, is particularly rich in these bioactive compounds due to its concentration of peel and pulp remnants, which are known to have higher phenolic content than fruit juice itself. This concentration of bioactive compounds in pear pomace enhances its potential as a functional food ingredient with significant health benefits, including the inhibition of MMP-9 activity. 

Furthermore, the consistent inhibitory effect observed across different granulometries suggests that the processing of pear pomace into varying particle sizes does not diminish its bioactive potential.

When comparing the MMP-9 inhibitory activity of pear pomace to clinical therapeutics, it is noteworthy that several synthetic MMP inhibitors, such as batimastat (BB-94) and marimastat (BB-2516), have been developed and tested in clinical settings for their ability to inhibit MMPs, including MMP-9 [36]. These inhibitors have shown efficacy in reducing tumor growth and metastasis in preclinical studies; however, their clinical success has been limited due to issues such as musculoskeletal toxicity and lack of specificity [36]. The natural inhibitors present in pear pomace, being part of a dietary intervention, may offer a safer and more sustainable alternative, as they are less likely to cause severe side effects and can be integrated into regular diets.

### 3.4. Identification and Quantification of Amino Acids Using UPLC-PDA-Q/TOF-MS

The amino acids analyzed using the UPLC-PDA-Q/TOF-MS method, along with their corresponding retention times, maximum absorption wavelengths, and specific parent and daughter ions ([M + H] + *m*/*z*), are presented in Table 2 and Table 3. Different particle size fractions of PPF 21 free amino acids were identified and quantified, including nine essential (EAAs) and five conditionally essential amino acids (CEAAs) for the human body. The total sum of amino acids ranged from 82.8 (in the fraction with particles of 180 µm) to 155.8 mg/100 g dw (in the fraction with particles of 53 µm), including EAAs and CEAAs). The correlation between these two groups of amino acids was high (r = 0.82).

Mahammad and co-workers previously reported the amino acid composition in seeds and pulp of pear fruits (*Pyrus communis*), where leucine (Leu), isoleucine (Ile), methionine (Met), phenylalanine (Phe), lysine (Lys), threonine (Thr), valine (Val), and histidine (His) were the EAAs identified [37]. As CEAAs, the same authors identified glycine (Gly), tyrosine (Tyr), arginine (Arg), cysteine (Cys), aspartic acid (Asp), glutamic acid (Glu), serine (Ser), and alanine (Ala) as non-essential amino acids (NEAAs). The same authors reported a higher content of EAAs and CEAAs in pear seeds (26 mg/100 g dw) than in pulp (14 mg/100 g dw) [37]. In this sense, we expected to have higher amino acid content in the fractions with higher particle sizes, which are mainly constituted by seeds and peel. However, EAAs exist in considerably higher amounts in fractions under 75 µm (around 96 mg/100 g dw) while in fractions with particles over 710 µm, the EAAs content was around 76 mg/100 g dw. This could be because the EAA and CEAA composition varies between cultivars, as has already been demonstrated by several authors [38]. The ratio of EAAs to CEAAs ranged from 0.5 (in fractions with particle sizes equal to or higher than 710 µm) to 0.6 (in fractions with particle size under 53 µm). The most abundant CEAA was proline (Pro), representing 61.4% of all CEAAs and 11.7% of all amino acids. 

The proline values varied between 59.3 and 63.6 mg/100 g dw, and different pear cultivars have a wide range of proline contents [39]. Cheongbae pear was reported to have a notably higher proline content and was identified as one of the disease-resistant cultivars [40]. Proline plays a vital role in supporting plant growth and defense mechanisms, acting as a key factor in the formation of numerous cell wall proteins. It serves as an essential component that accumulates within plants, offering benefits both during stressful situations and under normal, non-stressful conditions. As a beneficial solute, proline contributes significantly to the overall resilience and well-being of plants [41]. This may elucidate the significant elevation of proline levels observed in Cheongbae pear [41].

The nutritive value of the plants/vegetable/fruits protein quality is usually assessed by comparing its essential amino acids content with the reference standard ideal protein set using FAO/WHO/UNU [42]. The results of the amino acid analysis (Table 4) showed that the pomace contained some of the essential amino acids needed, although in lower amounts when compared to reference values [37]. The exception exists for isoleucine and valine, existing above the reference values (four-fold higher). Sulfur-containing amino acids such as methionine are still the most limiting amino acid. However, in comparison with the reference standard for ideal protein, the value for leucine content of PPF is considerably lower than in seeds, being under the recommended amino acid requirements (6.6 g/100 g protein) for infants. However, isoleucine content is considerably higher in PPF, being significantly above the reference value of 2.8 mg/100 g dw.

NEAAs were the most representative amino acids found in the PPF, accounting for 69 to 73% of the total amino acids present. The contents of individual NEAAs in the different fractions of PPF varied significantly, but the dominant ones—asparagine (Asn) and aspartic acid (Asp)—represented 22% (Asn) to 35% (Asp) of total amino acids. Yim and co-workers also found aspartic and glutamic acids as the major amino acids identified in different pear cultivars, ranging from 1151 to 6785 mg/100 g dw and 402 to 1009 mg/100 g dw, for aspartic acid and glutamic acid, respectively [40]. Chen and co-workers (2007) reported glutamic acid as the major amino acid among free amino acids with respect to the major pear cultivars [39]. In all fractions, glutamic acid (Glu), alanine (Ala), γ-amino-n-butyric acid (GABA), ornithine (Orn), and homocysteine (Cys) were also detected in significant amounts. The relatively high and diverse content of Asn, Asp, and Ala in the PPF fractions should be considered in the context of the risk of Maillard reactions causing the formation of potentially toxic compounds with negative health effects (acrylamide, hydroxymethylfurfural, heterocyclic amines, furans) [43]. 

### 3.5. Identification and Quantification of Carotenoids Using UPLC-PDA

The quantification of carotenoids was determined based on the UPLC-PDA analysis and standard curves, and the results are displayed in Table 5. When available, compounds were compared with authentic standards (their retention times, PDA profiles, and MS fragmentation spectra). If standards were not available, PDA profiles were compared with literature data.

Carotenoids are synthesized via various organisms including bacteria, algae, fungi, and plants. However, animals lack this ability and must acquire carotenoids from their diet [44]. These compounds are commonly found in colorful fruits and vegetables. For instance, carrots, apricots, pumpkins, and sweet potatoes are rich sources of α-carotene and β-carotene. Tomatoes and watermelons contain lycopene, ζ-carotene, β-carotene, phytofluene, and phytoene. Other fruits like mangoes, papayas, and vegetables such as spinach, peaches, and prunes provide lutein, zeaxanthin, α-, β-, and ζ-carotene, along with β-cryptoxanthin. The health benefits of carotenoids are substantial, including a reduced risk of cancer and improved immune system function. Research confirms their positive impact on metabolism regulation, decreased body fat deposition, and consequent weight reduction through daily consumption [19].

Results from Table 5 show that there is a decrease in total carotenoid content with a decrease in particle size for almost half the amount observed for fractions with particle sizes above 180 µm (around 21.9 mg/100 g dw). Our research also indicated that xanthophyll concentration ranged from 35% (for the fraction with particle size of 710 µm) to 44% of the total carotenoids (for the fraction with 180 µm). Again, as for total carotenoids, the amount of xanthophylls decreases with the particle size. The major identified xanthophyll was lutein, ranging from 3.9 to 8.4 mg/100 g dw, being lower in fraction with particle size under 53 µm. While lutein is the most abundant xanthophyll found in fruits and vegetables [45], zeaxanthin exists in much lower contents (only 4.0–6.5% of all carotenoids). Among the carotenoids identified, β-carotene is the predominant one, accounting for 55–64% of all carotenoids being most abundant in fractions with particle sizes over 75 µm. Overall, carotenes (β-carotenes and isomers) are more abundant than xanthophylls, representing 55–64.5% of all isoprenoids. However, as reported by Heinonen and co-workers [46], in fresh pear, lutein content is much higher (0.110 mg/100 g dw) than the β-carotene content (0.017 mg/100 g dw). In the PPF analysis, the differences are not as evident as in the fresh fruit [30], and both lutein and β-carotene contents, excluding its isomers, are around 36% of all isoprenoids identified.

Carotenoids play a crucial role in the body’s antioxidant defense system, alongside tocopherols, and that is why tocopherols and tocotrienols were found to be worth investigating.

### 3.6. Analysis of Tocopherols and Tocotrienols Using UPLC-FLR

Vitamins are vital phytochemicals essential for human health, with tocochromanols like tocopherol and tocotrienol isomers being lipid-soluble molecules represented by α, β, γ, and δ forms. These compounds, especially α-tocopherols, have distinct physiological functions and bioactive potentials, such as antioxidant, hypocholesterolemic, anticancer, and anti-inflammatory properties. They play a crucial role in protecting against degenerative diseases like Alzheimer’s and Parkinson’s disease [47]. Dietary intake serves as the primary source of tocochromanols, which are found in various plant parts such as roots, stems, tubers, flowers, and leaves [48]. Cereals, vegetables, nuts, seeds, oils, and some fruits are particularly rich sources [48]. Vitamin E, comprising tocopherols (TF) and tocotrienols (TT), acts as a potent antioxidant, safeguarding against cellular aging and environmental damage. It is predominantly present in the α-T form in plant tissues, except in seeds, where α- or γ-isomers prevail. Tocotrienols, more abundant in seeds, significantly contribute to antioxidant defense [49].

**Figure 3:**
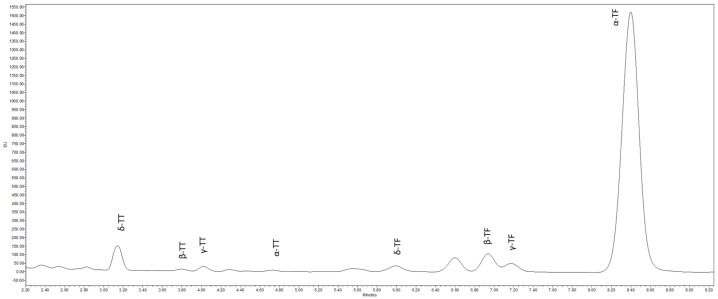
UPLC-FLR chromatogram (at the excitation/emission wavelength of 290/330 nm) of tocopherols and tocotrienols identified in the PPF fractions. TT: tocotrienols; TF: tocopherols.

Figure 3 illustrates a typical chromatographic profile of all tocopherol analogs detected in PPF, encompassing α-, β-, γ-, δ- tocopherol and tocotrienol homologs. As outlined in Table 6, tocopherols are notably more abundant compared to tocotrienol isomers, except for δ-TT in the fraction with particles over 710 µm (103.2 µg/100 g dw), β-TT, and γ-TT for fractions with particles between 180 µm < PS ≤ 710 µm, where TF levels are higher (177.5 and 132.4 µg/100 g dw, respectively). Moreover, the composition ratio of tocopherol to tocotrienol isomer varies significantly (*p* ≤ 0.05) among different fractions.

The α-TF isomer was quantified in all PPF samples as predominant (341.8–582.5 µg/100 g dw), particularly higher for fractions with particles over 180 µm, representing approximately 79% of all identified TF and 40% of all identified TT. This differs from reported data for pear seeds [50], where γ-TF is the most abundant isomer, accounting for 87% of all tocochromanols found specifically in seeds. Literature suggests that γ-tocopherol is the predominant isomer in seeds and their oils, exceeding 70%, whereas α-TF predominates in leaf tissues (>90%) [51]. The dominance of α-T indicates the presence and activity of the complete tocopherol pathway in plants, as α-T is regarded as the final step in tocopherol biosynthesis [52]. The remaining isomers, including β-, γ-, δ-TF, and TT, were comparatively lower, with γ-  >  β- > δ- for TT and β-  > γ- >  δ- for TF. Notably, the highest level of γ-TT was observed in the fraction with particles around 180 µm (132.4 µg/100 g dw), while, in other fractions, these contents were approximately 30 µg/100 g dw. The presence of γ-tocopherol is significant for plants as it acts as an antioxidant, potentially possessing equal or superior antioxidant properties to α-TF [53]. Foods rich in γ-tocopherol, such as nuts, are believed to reduce the risk of cardiovascular diseases [54].

The β-TT content was measured across all fractions, ranging from 4.5 to 22.9 μg/100 g dw, for particles over 710 µm and under 75 µm, respectively. Regarding TF, while the β-isomer was quantified around 71 μg/100 g dw, there was a notable decrease for the γ- and δ-isomers (78.6 and 7.5 μg/100 g dw, respectively). Literature reports vary in α-TF content in plants. Some studies indicate high levels of α-TF in certain plant parts, such as leaves of *Sauropus androgynus* and *Citrus hystrix*, shoots of *Ipomoea* batatas, *Carica* papaya, or fruits of red *Capsicum annum* [55]. Other research evaluated α-TF in various *Salvia* species, finding differing levels but consistent conditions or absence of damage symptoms [56].

In tocotrienols, the dominant isomer varied among granulometric fractions: for particles over 710 µm, δ-TT was the most abundant, comprising 46% of all identified TT; for particles between 710 < PS ≤ 180 µm, β- and γ-TT predominated (51% and 38% of all identified TT, respectively); for particles under 75 µm, α-TT was the most abundant, constituting approximately 57% of all TT. This variation may stem from the prevalence of certain isomers in specific plant tissues, such as γ-form in seeds and α-isomer in lesser amounts. Similar trends were noted in apricot and peach seeds, where the γ-isomer dominated (>60%), indicating incomplete conversion, as the expected end product, α-tocotrienol, was not predominant. Despite their low concentrations, tocotrienols exhibit potent antioxidant, anticancer, hypoglycemic, hypolipidemic, and cardioprotective activities compared to common tocopherol forms [54].

### 3.7. Identification and Quantification of Phenolic Compounds and Polymeric Procyanidins Using UPLC-PDA

The distribution of phenolic compounds in PPF fractions shows a clear dependency on particle size (Table 7). Phenolic concentrations vary widely, with the highest levels found in the fraction with particles sized between 710 µm and 180 µm, which had 660.1 mg/100 g dry weight (dw). This fraction also exhibited the greatest concentrations of flavonoids (636.3 mg/100 g dw) and polymeric procyanidins (514.8 mg/100 g dw). In contrast, the smallest particle size fraction (under 53 µm) had the lowest concentrations of these compounds, with phenolic compounds at 536.9 mg/100 g dw. The observed variation in phenolic content among different particle sizes can be linked to the anatomical distribution of phenolic compounds within the pear [10]. Larger particle sizes likely include more peel, which is richer in phenolics like anthocyanins, flavonols, catechins, and procyanidins. The peel’s higher surface area and exposure to external stresses might lead to an increased synthesis of these compounds as part of the fruit’s defense mechanism. Conversely, smaller particle fractions, containing more core material, show lower levels of these compounds because the core predominantly contains hydroxycinnamic acids and fewer of the phenolics associated with the peel [10].

When comparing the polyphenolic content in pears with other stone fruits, pears exhibit similar phenolic profiles to apples, with polyphenolic concentrations ranging from 1654.8 to 5314.1 mg/kg dw [50]. This similarity suggests that pears, like apples, have significant nutritional and antioxidant potential, particularly in terms of phenolic content. The variability in concentrations between different studies might be due to differences in pear varieties, growing conditions, and extraction methods.

Polymeric procyanidins are notably the most abundant flavonoids identified across all particle sizes, with concentrations ranging from 406.2 mg/100 g dw in the smallest particles to 514.8 mg/100 g dw in the 710 µm to 180 µm fraction. Procyanidins are significant because they are known to interact with cell wall polysaccharides such as pectin [57,58]. This interaction might influence the mechanical properties of the pear tissue and affect the bioavailability and extractability of these compounds during processing. The high concentration of procyanidins aligns with findings that procyanidins are major polyphenols in pear pomace and are crucial for the pomace’s antioxidant properties [59]. Among the identified phenolic acids, chlorogenic acid was the most prevalent, with concentrations ranging from 15.3 to 23.6 mg/100 g dw. Chlorogenic acid’s high levels highlight its importance in the phenolic profile of PPF. Known for its antioxidant activity, chlorogenic acid contributes to the overall health benefits associated with pear consumption. Its abundance further supports the potential use of PPF as a functional ingredient in food products aimed at enhancing antioxidant intake [60].

The significant levels of phenolics in larger particle fractions of PPF suggest that these fractions could be particularly valuable for industrial applications that aim to harness the antioxidant properties of pear by-products. This could include their use as natural antioxidants in food preservation, functional food ingredients, or supplements. The findings also underscore the potential health benefits of consuming products derived from pear pomace, especially those that retain the peel.

### 3.8. Lipophilic Extracts Composition Using GC-MS

As the advantageous characteristics of metabolites continue to be recognized, the fascination with metabolite profiling is steadily increasing. Presenting information about the metabolite profile of different fractions of the pear pomace is a piece of useful knowledge, especially in light of waste recovery and resource scarcity. Therefore, a semi-quantification GC-MS profile aids in the characterization of the PPF. The non-polar identified metabolites identified using GC-MS (Table 8) are divided into three main groups: sugars, sugar alcohols, and fatty acids. It is important to highlight the availability of potentially active substances in the pear pomace, as they are often regarded as food waste.

Saturated and unsaturated fatty acids are the most abundant compounds in all the PPF fractions analyzed, corresponding to 48–69% of all identified compounds in the dichloromethane extracts. Hexadecanoic acid and 9,12-(*Z, E*)-octadecadienoic acid were the most representative compounds in this class, corresponding to around 40% and 30% of all fatty acids, respectively. 9-(*Z*)-Octadecenoic acid was also quite abundant, corresponding to 20% of all fatty acids. Although in smaller amounts, tetradecanoic, octadecanoic, docosanoic, and tetracosanoic acids were also identified. Fractions with higher particle sizes (over 180 µm) show higher fatty acid content which may be explained by their composition, being enriched with peel and seeds [61].

Sugar alcohols constituted the second most abundant class of compounds, representing between 16% and 45% of all identified compounds in the dichloromethane extracts of PPF, being higher for fractions with lower particle sizes. Fructose and glucose (both isomers) represent 80–95% of all sugar alcohols. Non-polar compounds as sugars, namely, sorbitol and *myo*-inositol, constituted 4–20% of all compounds. In fact, and as reported in the literature, because of their unique composition of fiber, sorbitol, and fructose, pears have the potential to play an important role in regulating normal bowel function [62].

### 3.9. Correlation between the Composition of PPF and Its Bioactive Potential

Tocopherols and tocotrienols are recognized for their antioxidant properties, believed to inhibit lipid peroxidation in plant membranes, with the effectiveness of their antioxidant system varying based on stress levels [63]. The total content of tocopherols and tocotrienols in PPF showed a moderate correlation with their antioxidant activity (ranging from 0.16 to 0.72) as per ABTS and ORAC assays. Interestingly, in this investigation, the antioxidant activity of PPF exhibited a strong association with xanthophylls, carotenes, and carotenoids contents (ranging from 0.50 in ABTS assay to 0.64 in ORAC assay) and displayed a negative correlation with amino acids (ranging from −0.27 to −0.70), contrasting previous findings.

All the amino acids, except Asp, strongly correlate with anti-AChE activity (r ≥ 0.79), and the correlation with Asp was weak negative/neutral (r = −0.06). In the case of anti-BuChE activity, a moderate correlation with tocopherols (r ≥ 0.31), a strong correlation with xanthophylls (r ≥ 0.58), carotenes (r ≥ 0.82) and carotenoids (r ≥ 0.76), and a strong negative correlation with flavonoids, phenolic compounds, tocotrienols and some amino acids (Asp, GABA, Arg, His, Ile, Leu, and Phe) were found. Prior studies have highlighted the potential of vitamin E in slowing dementia progression and regulating signaling and gene expression. α-Tocopherol has shown promise in modulating pathways implicated in Alzheimer’s disease (AD). Supplementation with vitamin E has been observed to restore behavioral and cognitive functions, counteract oxidative stress and β-amyloid toxicity, and produce beneficial effects in AD animal models [64]. Aberrant levels of amino acids may indicate a pathogenesis linked to mild cognitive impairment and preclinical dementia. For instance, a prospective cohort study revealed that essential branched-chain amino acids (Val, Ile, and Leu) are associated with a reduced risk of dementia and Alzheimer’s disease [65]. In this study, these amino acids exhibited stronger correlations with the anti-AChE effect (ranging from 0.88 to 0.91). The elevated content of tocopherols, tocotrienols, and amino acids, correlated selectively with anti-AChE and/or anti-BuChE activity, underscores the significance of PPF in dietary approaches for treating neurodegenerative diseases.

## 4. Conclusions

This study evaluated the profile and potential applications of bioactive compounds in PPF across different granulometric fractions. The analysis included lipophilic compounds, phenolic compounds, amino acids, and tocols, alongside assessments of antioxidant potential, anti-acetylcholinesterase, anti-butyrylcholinesterase, and MMP-9 inhibitory activities. Key findings included the identification of flavonoids such as flavonols and flavan-3-ols, with polymeric procyanidins being the predominant phenolic compounds, and phenolic acids present as minor constituents. The study detected all tocopherol analogs, with α-tocopherol comprising over 60% of the total tocol content. Twenty-one amino acids were identified, including all nine essential amino acids, with asparagine and aspartic acid being the most abundant. Carotenoids like lutein and β-carotene were also found in smaller amounts. PPF fractions exhibited moderate AChE and BuChE inhibition (31% and 23%, respectively) compared to physostigmine salicylate and significant MMP-9 inhibitory potential, suggesting the use of PPF as a functional food ingredient to manage conditions associated with excessive MMP-9 activity, such as cancer, chronic inflammatory diseases, and age-related tissue degeneration. Natural phenolic compounds in PPF present a promising alternative to synthetic MMP inhibitors, offering a potentially safer and more accessible means of modulating MMP-9 activity through diet.

The study faced several limitations, including variability in the extraction method, which can lead to inconsistent results and challenges in comparing findings. Future research should focus on developing standardized extraction protocols for consistent measurement of bioactive compounds in PPF. Additionally, the stability of phenolic compounds and other bioactives during processing, storage, and utilization was not assessed. Investigating the stability under various conditions will be crucial to determine their long-term viability in food products. The bioavailability of PPF’s bioactive compounds also needs further exploration to understand their health impacts better. Furthermore, this study did not evaluate the functional and sensory attributes of PPF in food products, which are essential for consumer acceptance. Future research should explore these aspects to enhance PPF’s appeal and usability as a food ingredient.

In conclusion, while the study highlights the rich profile and potential applications of bioactive compounds in PPF, addressing these limitations through standardized extraction methods, stability studies, bioavailability research, and functional and sensory evaluations will further enhance PPF’s potential. This will enable its effective utilization as a sustainable and natural source of bioactive compounds, meeting the growing demand for health-promoting food ingredients and supplements.

## Figures and Tables

**Table 1:** Antioxidant activity (mmol Trolox/100 g dw) of the different PPF fractions, measured using ABTS and ORAC assays. The data shown are mean values (*n* = 3) followed by an alphabet letter (for comparison between the different granulometric fractions). Different letters mean significantly different results (Tukey’s HSD; *p* ≤ 0.05).

Particle Size Fractions (µm)	Antioxidant Activity (mmol Trolox/100 g dw)	
	ABTS	ORAC
710 µm	3.0 ± 0.09 ^a^	5.8 ± 0.15 ^a’^
180 µm	2.8 ± 0.03 ^a^	5.8 ± 0.20 ^a’^
75 µm	2.4 ± 0.09 ^a^	6.1 ± 0.32 ^a’^
53 µm	2.3 ± 0.07 ^a^	5.3 ± 1.30 ^a’^

**Table 2:** Essential amino acids’ composition (mg/100 g dw) of the different PPF fractions. The data shown are mean values (*n* = 3) followed by an alphabet letter (for comparison between the different granulometric fractions). Different letters mean significantly different results (*p* ≤ 0.05).

Granulation Degree	Essential Amino Acids (mg/100 g dw)									
	His	Thr	Lys	Met	Val	Ile	Leu	Phe	Trp	TOTAL
710 µm	11.4 ± 0.19 ^a^	3.2 ± 0.02 ^a^	0.4 ± 0.01 ^a^	0.2 ± 0.01 ^a^	11.6 ± 0.05 ^a^	7.5 ± 0.04 ^a^	1.9 ± 0.02 ^a^	0.5 ± 0.01 ^a^	1.3 ± 0.04 ^a^	37.9 ± 1.14 ^a^
180 µm	8.4 ± 0.13 ^b^	2.5 ± 0.02 ^b^	0.3 ± 0.01 ^b^	0.0 ± 0.01 ^b^	8.8 ± 0.05 ^b^	5.4 ± 0.03 ^b^	1.6 ± 0.02 ^b^	0.6 ± 0.01 ^b^	1.0 ± 0.05 ^b^	28.6 ± 1.00 ^b^
75 µm	16.1 ± 0.21 ^c^	4.2 ± 0.03 ^c^	0.6 ± 0.02 ^c^	0.3 ± 0.01 ^c^	14.7 ± 0.04 ^c^	9.9 ± 0.05 ^c^	2.5 ± 0.03 ^c^	0.9 ± 0.02 ^c^	1.5 ± 0.04 ^c^	50.7 ± 1.25 ^c^
53 µm	18.4 ± 0.27 ^d^	4.2 ± 0.03 ^c^	0.4 ± 0.01 ^a^	0.2 ± 0.01 ^a^	16.8 ± 0.03 ^d^	11.4 ± 0.07 ^d^	3.1 ± 0.03 ^d^	1.0 ± 0.03 ^d^	1.9 ± 0.06 ^d^	57.2 ± 1.34 ^d^

His: Hystidine; Thr: Threonine; Lys: Lysine; Met: Methionine; Val: Valine; Ile: Isoleucine; Leu: Leucine; Phe: Phenylalanine; Trp: Triptofane.

**Table 3:** Conditional essential amino acids composition (in mg/100 g dw) of the different PPF fractions. The data shown are mean values (*n* = 3) followed by an alphabet letter (for comparison between the different granulometric fractions). Different letters mean significantly different results (*p* ≤ 0.05).

Granulation Degree	Conditionally Essential Amino Acids (mg/100 g dw)					
	Arg	Gln	Gly	Pro	Tyr	TOTAL
710 µm	5.9 ± 0.03 ^a^	18.9 ± 0.31 ^a^	2.8 ± 0.02 ^a^	48.4 ± 0.25 ^a^	0.2 ± 0.01 ^a^	76.2 ± 0.63 ^a^
180 µm	5.1 ± 0.02 ^b^	14.9 ± 0.22 ^b^	1.8 ± 0.01 ^b^	32.2 ± 0.31 ^b^	0.2 ± 0.01 ^a^	54.3 ± 0.58 ^b^
75 µm	11.1 ± 0.07 ^c^	23.4 ± 0.28 ^c^	2.7 ± 0.01 ^c^	55.5 ± 0.27 ^c^	0.2 ± 0.02 ^a^	93.0 ± 0.83 ^c^
53 µm	11.8 ± 0.08 ^d^	23.7 ± 0.31 ^c^	3.0 ± 0.02 ^d^	60.0 ± 0.34 ^d^	0.2 ± 0.01 ^a^	98.6 ± 0.74 ^d^

Arg: Arginine; Gln: Glutamine; Gly: Glycine; Pro: Proline; Tyr: Tyrosine.

**Table 4:** Comparison of EAA composition of the seeds, pulp, and pomace of pears fruit with FAO/WHO/UNU reference values.

Amino Acid	Composition (mg/100 g dw)			FAO/WHO/UNU Reference Value **
	Seed	Pulp	Pomace (Mix)	
Ile	3.7	3.2	10.3	2.8
Leu	6.9	5.0	2.7	6.6
Lys	4.4	3.1	0.6	5.8
Met + Cys	2.7	1.3	0.3 *	2.5
Phe + Tyr	6.7	3.5	1.2	6.3
Thr	2.2	2.1	4.2	3.4
Val	3.9	3.0	15.9	3.5

Ile: Isoleucine; Leu: Leucine; Lys: Lysine; Met + Cys: Methionine + Cysteine; Phe + Tyr: Phenylalanine + Tyrosine; Thr: Threonine; Val: Valine. * Just methionine (Met) content. ** Source: FAO/WHO/UNU (1991).

**Table 5:** Identified and quantified carotenoids (mg/100 g dw) by classes: xanthophylls, carotenes, and total carotenoids of the different PPF fractions. The data shown are mean values (*n* = 3) followed by an alphabet letter (for comparison between the different granulometric fractions). Different letters mean significantly different results (*p* ≤ 0.05).

Granulation Degree	Carotenoids (mg/100 g dw)								
	Lutein	Zeaxanthin	Total Xanthophylls	β-Carotene	β-Carotene Isomer I	β-Carotene Isomer II	β-Carotene Isomer III	Total Carotenes	Total Carotenoids
710 µm	6.1 ± 0.47 ^a^	0.9 ± 0.15 ^a^	7.0 ± 0.26 ^a^	8.6 ± 0.21 ^a^	2.0 ± 0.12 ^a^	0.5 ± 0.09 ^a^	1.9 ± 0.28 ^a^	12.9 ± 0.24 ^a^	20.0 ± 0.54 ^a^
180 µm	8.4 ± 0.46 ^b^	1.3 ± 0.09 ^b^	9.7 ± 0.47 ^b^	7.5 ± 0.31 ^b^	2.2 ± 0.19 ^a^	1.0 ± 0.12 ^b^	1.4 ± 0.24 ^a^	12.1 ± 0.28 ^b^	21.9 ± 0.99 ^a^
75 µm	6.8 ± 0.37 ^a^	1.1 ± 0.10 ^a,b^	8.0 ± 0.46 ^c^	7.8 ± 0.47 ^b^	1.7 ± 0.18 ^a^	0.3 ± 0.04 ^c^	1.5 ± 0.14 ^a^	11.2 ± 0.36 ^c^	19.2 ± 0.48 ^a^
53 µm	3.9 ± 0.24 ^c^	0.7 ± 0.07 ^a,c^	4.6 ± 0.35 ^d^	3.6 ± 0.26 ^c^	1.6 ± 0.20 ^a^	0.4 ± 0.01 ^a^	0.6 ± 0.09 ^b^	6.2 ± 0.27 ^d^	10.8 ± 0.46 ^b^

**Table 6:** Identified and quantified tocopherols and tocotrienols (µg/100 g dw) of the different PPF fractions. The data shown are mean values (*n* = 3) followed by an alphabet letter (for comparison between the different granulometric fractions). Different letters mean significantly different results (*p* ≤ 0.05).

Granulation Degree	Tocopherols (TF) and Tocotrienols (TT) (µg/100 g dw)							
	Delta TT	Beta TT	Gamma TT	Alfa TT	Delta TF	Beta TF	Gamma TF	Alfa TF
710 µm	103.22 ± 0.86 ^a^	4.55 ± 0.20 ^a^	18.49 ± 0.18 ^a^	96.10 ± 0.82 ^a^	10.31 ± 0.22 ^a^	83.58 ± 0.67 ^a^	50.35 ± 0.48 ^a^	582.53 ± 1.58 ^a^
180 µm	9.02 ± 0.36 ^b^	177.54 ± 0.58 ^b^	132.42 ± 0.44 ^b^	28.03 ± 0.17 ^b^	4.39 ± 0.10 ^b^	68.08 ± 0.53 ^b^	45.55 ± 0.34 ^b^	795.4 ± 1.40 ^b^
75 µm	7.70 ± 0.36 ^b^	22.87 ± 0.04 ^c^	45.52 ± 0.19 ^c^	102.04 ± 1.94 ^c^	3.04 ± 0.07 ^c^	80.62 ± 0.66 ^c^	37.75 ± 0.50 ^c^	354.1 ± 1.15 ^c^
53 µm	20.68 ± 0.36 ^c^	14.58 ± 0.10 ^d^	44.34 ± 0.28 ^d^	93.96 ± 0.83 ^d^	12.59 ± 0.11 ^d^	53.73 ± 0.70 ^d^	60.58 ± 0.31 ^d^	341.84 ± 1.58 ^d^

TT: Tocotrienols; TF: Tocopherols.

**Table 7:** Identified and quantified phenolic compounds (mg/100 g dw) of the different PPF fractions. The data shown are mean values (*n* = 3) followed by an alphabet letter. Different letters mean significantly different results (*p* ≤ 0.05).

Granulation Degree	Phenolic compounds (mg/100 g dw)					
	Phenolic Acids	Flavonols	Flavan-3-ols	Polymeric Procyanidins	Total Flavonoids	Total Phenolic Compounds
710 µm	25.09 ± 0.14 ^b^	77.10 ± 1.02 ^a^	43.50 ± 0.40 ^a^	429.10 ± 2.70 ^a^	549.71 ± 2.75 ^a^	574.79 ± 2.79 ^a^
180 µm	23.80 ± 0.17 ^c^	80.50 ± 0.92 ^b^	41.03 ± 0.32 ^b^	514.82 ± 2.64 ^b^	636.35 ± 2.69 ^b^	660.15 ± 2.72 ^b^
75 µm	16.49 ± 0.11 ^d^	89.42 ± 1.04 ^c^	33.89 ± 0.47 ^c^	412.75 ± 2.31 ^c^	536.05 ± 2.34 ^c^	552.54 ± 2.36 ^c^
53 µm	16.40 ± 0.15 ^d^	80.73 ± 1.10 ^b^	33.64 ± 0.38 ^c^	406.16 ± 2.10 ^d^	520.53 ± 2.16 ^d^	536.93 ± 2.24 ^d^

**Table 8:** Composition of the different PPF dichloromethane extracts, in % of the chromatographic peak areas of the compounds detected using GC-MS. The data shown are mean values (*n* = 3) followed by an alphabet letter. Different letters mean significantly different results (*p* ≤ 0.05).

	Granulation Degree of the Different PPF			
**Chemical compounds**	710 µm	180 µm	75 µm	53 µm
**Sugars**	**19.8 ± 1.70 ^a^**	**13.4 ± 0.71 ^b^**	**3.4 ± 0.57 ^c^**	**6.0 ± 1.13 ^d^**
Sorbitol, 6-TMS ester	8.7 ± 0.06 ^a^	7.2 ± 0.52 ^b^	1.3 ± 0.07 ^c^	2.2 ± 0.01 ^d^
Myo-inositol, 6-TMS ester	11.1 ± 1.01 ^a^	6.2 ± 0.06 ^b^	2.1 ± 0.14 ^c^	3.8 ± 0.13 ^d^
**Sugar alcohols**	**21.6 ± 3.91 ^a^**	**15.1 ± 2.49 ^b^**	**40.6 ± 3.25 ^c^**	**32.0 ± 5.16 ^d^**
Fructose, 5-TMS ester	7.4 ± 0.14 ^a^	5.1 ± 0.03 ^b^	11.2 ± 1.12 ^c^	11.0 ± 2.65 ^c^
Fructose isomer, 5-TMS ester	4.7 ± 0.38 ^a^	6.2 ± 0.01 ^b^	9.4 ± 0.86 ^c^	12.6 ± 1.02 ^d^
Glucose, 5-TMS ester	2.3 ± 0.08 ^a^	1.6 ± 0.21 ^b^	5.3 ± 0.58 ^c^	3.4 ± 0.06 ^d^
Glucose isomer, 5-TMS ester	1.1 ± 0.03 ^a^	1.1 ± 0.14 ^a^	6.7 ± 0.09 ^b^	3.3 ± 0.41 ^c^
Sucrose, 8-TMS ester	3.1 ± 0.06 ^a^	0.2 ± 0.00 ^b^	1.9 ± 0.06 ^b^	1.0 ± 0.00 ^c^
Sucrose isomer, 8-TMS ester	1.0 ± 0.04 ^a^	0.9 ± 0.04 ^b^	6.1 ± 0.13 ^c^	0.7 ± 0.01 ^d^
**Saturated and unsaturated fatty acids**	**57.6 ± 7.37 ^a^**	**63.3 ± 8.82 ^a^**	**45.3 ± 8.30 ^a^**	**41.6 ± 7.41 ^a^**
Tetradecanoic acid, TMS ester	0.2 ± 0.02 ^a^	1.3 ± 0.06 ^b^	1.3 ± 0.11 _b_	0.9 ± 0.03 _c_
Hexadecanoic acid, TMS ester	22.3 ± 0.14 ^a^	19.6 ± 0.31 ^b^	22.6 ± 1.03 ^a^	21.0 ± 2.33 ^a,b^
9,12-(Z, E)-Octadecadienoic acid, TMS ester	20.9 ± 0.06 ^a^	22.9 ± 0.43 ^b^	9.9 ± 1.44 ^c^	7.4 ± 0.31 ^d^
9-(Z)-Octadecenoic acid, TMS ester	9.4 ± 0.21 ^a^	8.7 ± 1.03 ^a^	9.7 ± 0.07 ^a^	8.6 ± 0.51 ^a^
Octadecanoic acid, TMS ester	1.3 ± 0.04 ^a^	6.1 ± 0.06 ^b^	1.4 ± 0.11 ^a^	0.2 ± 0.04 ^c^
Docosanoic acid, TMS ester	0.9 ± 0.03 ^a^	0.8 ± 0.11 ^a^	0.3 ± 0.07 ^b^	1.3 ± 0.11 ^c^
Tetracosanoic acid, TMS ester	2.6 ± 0.13 ^a^	3.9 ± 0.07 ^b^	0.1 ± 0.01 ^c^	2.2 ± 0.45 ^a^
**Identified compounds**	**99.0 ± 3.95 ^a^**	**91.8 ± 2.33 ^b^**	**89.3 ± 2.30 ^b^**	**79.6 ± 1.42 ^c^**
Non-identified compounds	1.0	8.2	10.7	21.0
TOTAL	100.0	100.0	100.0	100.0

## Data Availability

Dataset available on request from the authors.
